# The Mormyrid Optic Tectum Is a Topographic Interface for Active Electrolocation and Visual Sensing

**DOI:** 10.3389/fnana.2018.00079

**Published:** 2018-10-01

**Authors:** Malou Zeymer, Gerhard von der Emde, Mario F. Wullimann

**Affiliations:** ^1^Department of Neuroethology/Sensory Ecology, Institute for Zoology, University of Bonn, Bonn, Germany; ^2^Biocenter, Department Biology II, Ludwig-Maximilians-Universität München, Munich, Germany

**Keywords:** mormyrid, electrosensation, *Gnathonemus petersii*, optic tectum, torus semicircularis, vision

## Abstract

The African weakly electric fish *Gnathonemus petersii* is capable of cross-modal object recognition using its electric sense or vision. Thus, object features stored in the brain are accessible by multiple senses, either through connections between unisensory brain regions or because of multimodal representations in multisensory areas. Primary electrosensory information is processed in the medullary electrosensory lateral line lobe, which projects topographically to the lateral nucleus of the torus semicircularis (NL). Visual information reaches the optic tectum (TeO), which projects to various other brain regions. We investigated the neuroanatomical connections of these two major midbrain visual and electrosensory brain areas, focusing on the topographical relationship of interconnections between the two structures. Thus, the neural tracer DiI was injected systematically into different tectal quadrants, as well as into the NL. Tectal tracer injections revealed topographically organized retrograde and anterograde label in the NL. Rostral and caudal tectal regions were interconnected with rostral and caudal areas of the NL, respectively. However, dorsal and ventral tectal regions were represented in a roughly inverted fashion in NL, as dorsal tectal injections labeled ventral areas in NL and vice versa. In addition, tracer injections into TeO or NL revealed extensive inputs to both structures from ipsilateral (NL also contralateral) efferent basal cells in the valvula cerebelli; the NL furthermore projected back to the valvula. Additional tectal and NL connections were largely confirmatory to earlier studies. For example, the TeO received ipsilateral inputs from the central zone of the dorsal telencephalon, torus longitudinalis, nucleus isthmi, various tegmental, thalamic and pretectal nuclei, as well as other nuclei of the torus semicircularis. Also, the TeO projected to the dorsal preglomerular and dorsal posterior thalamic nuclei as well as to nuclei in the torus semicircularis and nucleus isthmi. Beyond the clear topographical relationship of NL and TeO interconnections established here, the known neurosensory upstream circuitry was used to suggest a model of how a defined spot in the peripheral sensory world comes to be represented in a common associated neural locus both in the NL and the TeO, thereby providing the neural substrate for cross-modal object recognition.

## Introduction

The African mormyriform fish represent one of only two groups of teleostean weakly electric fish, the other one being the South-American gymnotiforms. These fish possess a weak electric organ capable of emitting electric organ discharges (EODs) that can be used for electrocommunication and electrolocation through the involvement of electroreceptor organs on their body surface (reviewed in Bell and Szabo, 1986; von der Emde, 1998; Wullimann and Grothe, 2013; see discussion for more information and citations). Thus, these fish can perform active electrolocation in complete darkness and orient in a three-dimensional environment at least as swiftly as other vertebrates do using vision (von der Emde, 1999, 2004, 2006; von der Emde and Schwarz, 2000, 2002; von der Emde et al., 2008, 2010). However, mormyrids also use their eyes for orienting and object recognition (Landsberger et al., 2008; Kreysing et al., 2012; Pusch et al., 2013a; Schumacher et al., 2017; see Discussion for more information and citations). This enables mormyrids potentially to create a central nervous representation of their environment using two image-forming sensory systems during object localization and discrimination.

Indeed, the mormyrid *Gnathonemus petersii* can be trained to differentiate between objects with identical volumes but different shapes using exclusively the visual system or the electrosensory system and later be tested for recognition of a learned object with the respective other sense with which the object in question has never been experienced before (Schumacher et al., 2016a,b, 2017). Thus, it was shown that cross-modal object recognition (or modality transfer) is possible for these fish (Schumacher et al., 2016a). However, the central nervous substrate for this process has not been established.

**FIGURE 1:**
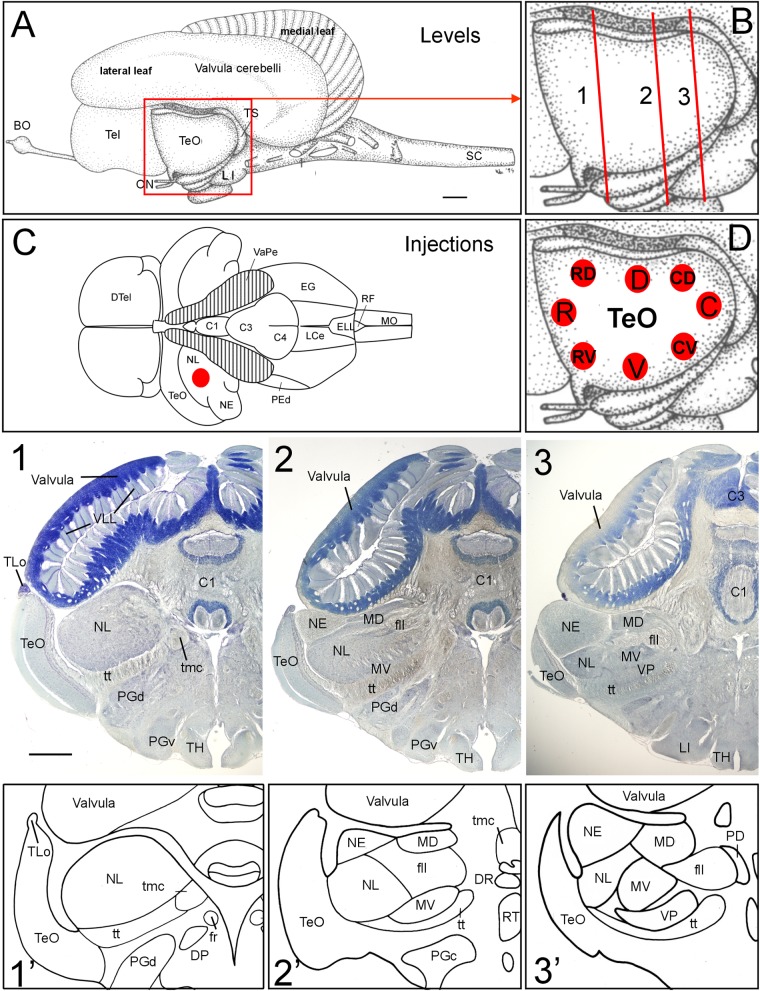
General brain neuroanatomy of *Gnathonemus petersii*, transverse brain levels of investigation and DiI injection sites. **(A)** Lateral view of the brain of the elephant-nose fish *G. petersii*. **(B)** Enlargement of optic tectum shows three levels documented below for tecto-toral interconnections. **(C)** Dorsal view of the brain of *G. petersii* with valvula cerebelli removed to show torus semicircularis and position of injection sites in the lateral toral nucleus (redrawn from Han et al., 2006). **(D)** Positions of DiI injections in two series of experiments with four quadrants each of optic tectum (R, rostral; C, caudal; D, dorsal; V, ventral; RV, rostroventral; RD, rostrodorsal; CV, caudoventral; CD, caudodorsal). **(1,2,3)** Row of Bodian-Cresyl stained sections of **(1)** anterior level of torus semicircularis with only the lateral toral nucleus, **(2)** mid-toral level with medioventral toral and exterolateral toral nuclei in addition and **(3)** caudal toral level with the ventroposterior toral nucleus in addition. **(1’,2’,3’)** Drawings highlight toral nuclei at levels shown in Bodian-Cresyl stains. Size bar in **(A)**: 1 mm, also applies to **(C)**. Size bar in **(1)**: 1 mm, also applies to **(2,3)**. See text for details. BO, bulbus olfactorius; C1, C3, C4, lobes of corpus cerebelli; DP, dorsal posterior thalamic nucleus; DR, rostrodorsal tegmental nucleus; DTel, dorsal telencephalon; EG, eminentia granularis; ELL, electrosensory lateral line lobe; fll, lateral longitudinal fascicle; fr; fasciculus retroflexus; LCe, lobus caudalis cerebelli; LI, lobus inferior; MD, mediodorsal nucleus of torus semicircularis; MO, medulla oblongata; MV, medioventral nucleus of torus semicircularis; NE, exterolateral nucleus of torus semicircularis; NL, lateral nucleus of torus semicircularis; ON, optic nerve; PD, dorsal perilemniscal part of nucleus lateralis valvulae; PEd, dorsal pre-eminential nucleus; PGc, PGd, PGv caudal, dorsal, ventral preglomerular nuclei; RF, rhomboid fossa; RT, rostral tegmental nucleus (of Grover and Sharma, 1981); SC, spinal cord; Tel, telencephalon; TeO, tectum opticum; TH, tuberal hypothalamus; TLo, torus longitudinalis; tmc, mesencephalo-cerebellar tract; TS, torus semicircularis; tt, toro-pre-eminential tract; VaPe, valvular peduncle (cut); VLL, valvular leaflets; VP, ventroposterior nucleus of torus semicircularis.

In line with these amazing sophisticated behaviors, mormyrids exhibit a brain-body weight ratio comparable to mammals and birds (Jerison, 2001). This is largely, but not exclusively, due to the size of the cerebellum. The German neuroanatomist Erdl (1846) noted - to our knowledge for the first time - the large size and rostral extent of the cerebellar valvula (see **Figure 1A**) and interpreted it as the telencephalon. The true cerebellar nature of the valvula was only established by a series of early 20th century neuroanatomists such as Franz (1912, 1913, 1921), Stendell (1914a,b,c), Berkelbach van der Sprenkel (1915), and Suzuki (1932). Mormyrid brain histology ranges among the most magnificently differentiated ones within vertebrates, as reported by Rudolf Nieuwenhuys and collaborators (Nieuwenhuys, 1963; Nieuwenhuys and Nicholson, 1969a,b; Nieuwenhuys et al., 1974; Meek et al., 1986a, 1989).

In parallel, the functional neuroanatomy of the mormyrid brain was brought forward initially by Curtis Bell, Thomas Finger, Thomas Szabo and Johannes Meek (see Discussion). This body of work established the general ascending electrosensory pathway from the hindbrain primary electrosensory lateral line lobe (ELL) through midbrain (torus semicircularis) into diencephalic and finally telencephalic (pallial) levels, which parallels much of what is known in all aquatic anamniote vertebrates for the mechanosensory lateral line pathway (review: Wullimann and Grothe, 2013). However, also distinct mormyrid specializations such as the heavy involvement of the valvula (and corpus) cerebelli in this ascending circuitry were revealed (Finger et al., 1981; Meek et al., 1986a,b). The midbrain optic tectum (Lázár et al., 1984) and lateral nucleus of the torus semicircularis (Hollmann et al., 2016) contain neural representations of the sensory periphery, one visual, the other electrosensory. Thus, these two structures are prime candidates for the neural substrate for cross-modal object recognition. This also applies to the valvula cerebelli, because it also has reciprocal connections with the lateral toral nucleus whereas the valvula has apparently only efferent connections to the optic tectum. The diencephalic dorsal preglomerular nucleus is another candidate because both tectum and lateral toral nucleus project topographically to it (see Discussion). Although topography has not been addressed in the mormyrid pallium, there are non-overlapping sensory fields for audition, lateral line, electrolocation and vision (Prechtl et al., 1998; von der Emde and Prechtl, 1999) speaking for parallel processing of sensory information at the highest central nervous level. Considering all this, the question arises where in the brain the mormyrid electrosense and vision might interact.

Unfortunately, the role of the optic tectum in electrosensory circuitry, in particular in the electrolocation pathway, has not been elucidated in mormyrids. In a review of 1986, Bell and Szabo only claimed cursorily that there is a topographic projection from the lateral toral nucleus to the ispilateral optic tectum without giving detail. Because the midbrain is the brain level where visual and electrosensory information comes together, we wanted to know whether the neuroanatomical connections between these two structures maintain topography. Therefore, we focus in this study on the interconnections of the mormyrid lateral nucleus of the torus semicircularis and the optic tectum by establishing their detailed neuroanatomical connections to support their possible role in object localization and recognition. Our tracer injections into various tectal quadrants and two lateral toral nucleus injections show that topography is maintained among the two midbrain structures and that they qualify as possible substrate for cross-modal sensory interactions. Moreover, we develop a model of how the sensory periphery sensed by the electrosensory and the visual systems might converge in the lateral toral nucleus.

## Materials and Methods

### Study Animals and Rearing Conditions

Sixteen specimens of the African weakly electric fish *Gnathonemus petersii* of undetermined sex with a standard length of 7–12 cm were obtained from a local dealer (Aquarium Glaser, Rodgau, Germany). Fish were maintained in a comfortable environment, housed in groups of 5 -15 individuals per 200 liter tank, in an accredited animal facility respecting European guidelines conforming Directive 2010/63/EU. The light-dark cycle was set to 12:12 h; the temperature (25° ± 1°C) and the conductivity (100 ± 5 μS/cm) of the water were kept constant. Fish were fed with defrosted red bloodworms (Chironomidae) every weekday. The experiments were approved by the state authority (Landesamt für Natur, Umwelt und Verbraucherschutz Nordrhein-Westfalen, LANUV, 84-02.04.2015.A444).

### Animal Perfusions and Fixation

Before fixation, fish were anesthetized in a 0.2 g/l tricaine methane sulfonate (MS-222, Acros Organics, Geel, Belgium) solution. Afterward, fish were intrabuccally ventilated with MS-222 solution at a euthanizing concentration of 0.1 g/l. The perfusion via the heart with 50 ml teleost Ringer solution (Wolf, 1963) was followed by 50 ml 4% paraformaldehyde (PFA, Roth, Karlsruhe, Germany) in Sörensen phosphate buffer (pH 7.38). After removing the brains, they were fixed in 4% PFA at 4°C for 48 h.

### DiI Tracing Method

After fixation, fine crystals of the carbocyanine dye 1,12′-dioctadecyl-3,3,32,32-tetramethylindo-carbocyanine perchlorate [D282, ‘DiI′; DiIC18(3), invitrogen, Molecular Probes, Inc., Eugene, OR, United States] were injected into different areas of the optic tectum (TeO; 12 cases; including four repeats; **Figure 1D**) or in the lateral nucleus of torus semicircularis (NL; 2 cases; **Figure 1C**). Before the injection into the NL, either part of the ipsilateral valvula cerebelli or the TeO was removed to uncover the NL. DiI crystals were inserted into the specific brain areas with a fine needle and afterward sealed with 4% agar-agar (Roth, #5210) to ensure that the DiI crystals remained in place.

The brains were incubated in 4% PFA at 37°C in an incubation chamber for 37–43 or 69–72 days. After incubation, the agar drop on the injection was removed and the brain was embedded in 4% agar-agar. The embedded brain was then glued to a base followed by cutting on a vibratome (Leica VT1000S) at 60–80 μm. The brain sections were mounted on gelatinized glass slides (Diagonal, Münster; #02 1102) and coverslipped with fluorescent mounting medium Vectashield (Vector Laboratories Inc.).

### Bodian-Cresyl Violet Method

For additional illustration we used some photographs from an archived series of transverse brain Nissl and Bodian silver-protein stained sections of *G. petersii* prepared decades ago by the senior author. Methodological details may be found in Wullimann and Northcutt (1990).

### Microscopy

The brain sections were photographed using a light/fluorescence microscope (Nikon Eclipse 80i; Nikon Instruments Inc.,) with a Nikon Digital Sight DSU1 Photomicrographic Camera (Nikon Instruments Inc.,) and LUCIA-G5 software. The microscope was equipped with Nikon Plan UW 0.06 (2x), Plan Fluor 109/0.30 (10x) and Plan Fluor 209/.0.50 (20x) objectives. Additionally, close-up images were taken with a Leica TCS SP-5 confocal laser-scanning microscope (Leica Microsystems).

All images were taken as monochrome pictures, slightly adapted for brightness and contrast with either Corel PHOTO-PAINT 9.0 or ImageJ and mounted into figures with Corel DRAW 9.0 (Corel Corporation).

## Results

### General Neuroanatomy and Tracer Injection Sites

The brain of the elephant-nose fish *Gnathonemus petersii* (Mormyridae) is characterized by an enormously enlarged valvular part of the cerebellum (Meek et al., 2008; Shi et al., 2008; Zhang et al., 2011; **Figure 1**). Instead of its usual position within the tectal ventricle seen in other teleosts, the mormyrid valvula resides dorsal to the two other cerebellar divisions, the vestibulolateralis lobe (Campbell et al., 2007), which consists of the medially located lobus caudalis (LCe) and the lateral eminentia granularis (EG) (**Figures 1C**, **8D,G**), and the corpus cerebelli (lobes C1 through C4 shown in **Figures 1C**, **8G**) (Han et al., 2006). What is more, the valvular anterior extent surpasses the midbrain and enters the area dorsal to diencephalon and telencephalon (**Figure 1A**). Additional and even greater enlargement of the cerebellar surface is reached by the outer valvular leaf folding over an inner one. Furthermore, uncounted fine leaflets of cerebellar molecular layer tissue (VLL in **Figure 1-1**) are formed in each leaf.

**FIGURE 2:**
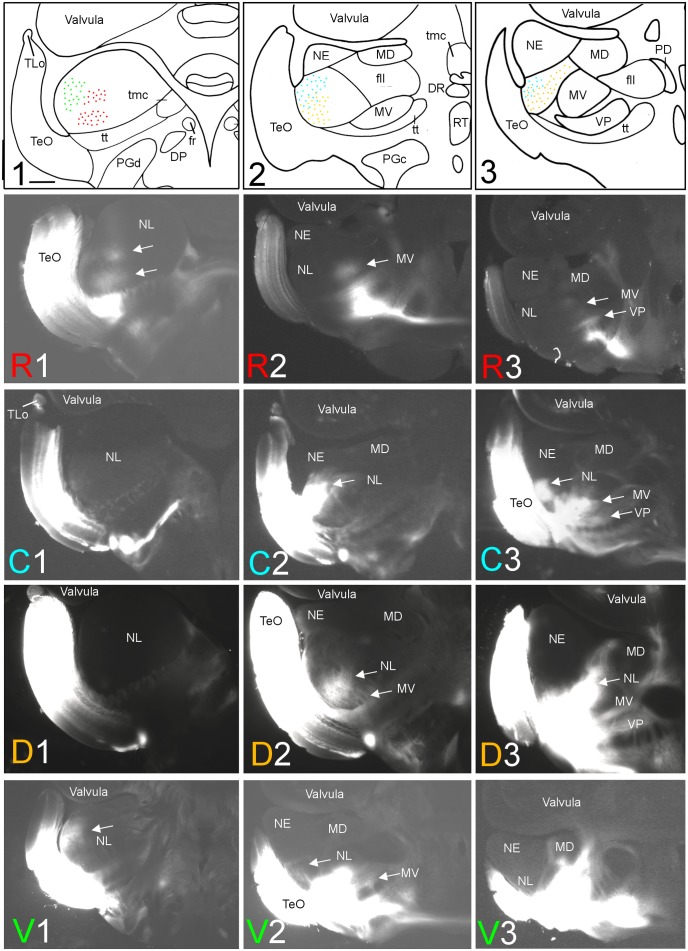
Topography of tecto-toral interconnections in *Gnathonemus petersii*. **(1–3)** Drawings of the three levels of the torus semicircularis documented in four rows below **(R1 – R3/C1 – C3/D1 – D3/V1 – V3)** each row showing toral connections after one of four tectal DiI injections (R, rostral; C, caudal; D, dorsal; V, ventral). **Arrows** point to labeled fields. All connections are ipsilateral. Note evident topographical relationship between lateral toral nucleus and optic tectum, also schematically visualized in top drawings 1–3 with corresponding colors. Size bar in **(1)**: 0.5 mm, applies to all panels. See text for details. DP, dorsal posterior thalamic nucleus; DR, rostrodorsal tegmental nucleus; fr, fasciculus retroflexus; fll, lateral longitudinal fascicle; MD, mediodorsal nucleus of torus semicircularis; MV, medioventral nucleus of torus semicircularis; NE, exterolateral nucleus of torus semicircularis; NL, lateral nucleus of torus semicircularis; PD, dorsal perilemniscal part of nucleus lateralis valvulae; PGd, PGc, dorsal, caudal preglomerular nuclei; RT, rostral tegmental nucleus (of Grover and Sharma, 1981); TeO, tectum opticum; TLo, torus longitudinalis; tmc, mesencephalo-cerebellar tract; tt, toro-pre-eminential tract; VP, ventroposterior nucleus of torus semicircularis.

**FIGURE 3:**
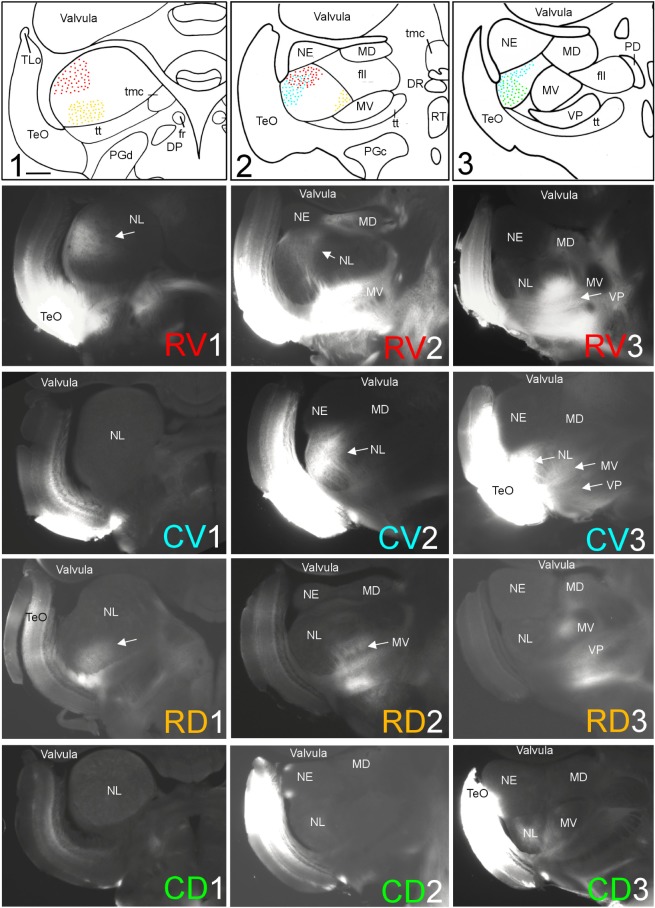
Topography of tecto-toral interconnections in *Gnathonemus petersii*. **(1–3)** Drawings of the three levels of the torus semicircularis documented in four rows below **(RV1 – RV3/CV1 – CV3/RD1 – RD3/CD1 – CD3)** each row showing toral connections after one of four tectal DiI injections (RV, rostroventral; CV, caudoventral; RD, rostrodorsal; CD, caudodorsal). **Arrows** point to labeled fields. All connections are ipsilateral. Note evident topographical relationship between lateral toral nucleus and optic tectum, also schematically visualized in top drawings 1–3 with corresponding colors. Size bar in **(1)**: 0.5 mm, applies to all panels. See text for details. DP, dorsal posterior thalamic nucleus; DR, rostrodorsal tegmental nucleus; fr, fasciculus retroflexus; fll, lateral longitudinal fascicle; MD, mediodorsal nucleus of torus semicircularis; MV, medioventral nucleus of torus semicircularis; NE, exterolateral nucleus of torus semicircularis; NL, lateral nucleus of torus semicircularis; PD, dorsal perilemniscal part of nucleus lateralis valvulae; PGd, PGc, dorsal, caudal preglomerular nuclei; RT, rostral tegmental nucleus (of Grover and Sharma, 1981); TeO, tectum opticum; TLo, torus longitudinalis; tmc, mesencephalo-cerebellar tract; tt, toro-pre-eminential tract; VP, ventroposterior nucleus of torus semicircularis.

A consequence of this massive valvular hypertrophy is the lateroventral displacement of the midbrain optic tectum (TeO; **Figure 1A**). Furthermore, transverse Bodian-Cresyl violet stained sections (**Figures 1-1**–**3**, comparable drawings shown in **Figures 1-1**’–**3**’) reveal that the second alar midbrain division, the torus semicircularis, includes five large nuclei (lateral, exterolateral, mediodorsal, medioventral and ventroposterior nuclei), which are all involved in hair cell related sensory circuitries. We chose three toral levels (**Figure 1B**) for documenting the neuroanatomical connections between torus semicircularis and optic tectum, namely a rostral level, an intermediate one and a caudal one, indicated with Arabic numbers 1 to 3 in **Figure 1** and all subsequent figures. We will first report the results after DiI Injections into various optic tectum quadrants of *G. petersii* (**Figure 1D**) followed by a description of connections after injections of DiI into the dorsal part of the lateral toral nucleus (NL, **Figure 1C**).

The neuroanatomical terminology follows Nieuwenhuys (1963) and Meek et al. (1986a, 1989) with some changes introduced for the preglomerular region by Wullimann and Northcutt (1990).

### Injections Into Optic Tectum

We applied small solid particles of DiI with a fine needle into either rostral (R), caudal (C), dorsal (D) or ventral (V) tectal quadrants of PFA fixed brains of *G. petersii* (see **Figures 1D**, **2**). In a second set of experiments, we injected four tectal quadrants shifted by 45°, i.e., RV: rostroventral injection, CV: caudoventral injection; RD: rostrodorsal injection, CD: caudodorsal injection (see **Figures 1D**, **3**). We will first focus on the lateral toral nucleus (NL), followed by the remaining toral nuclei, then continue with additional tectal connections, and conclude with tectal connections with the valvula cerebelli. Of note, we only saw ipsilateral - but not contralateral – label in toral nuclei.

#### Connections With the Lateral Toral Nucleus

After rostral tectal injections, antero- and retrograde label was only observed in the anteroventral aspect of the lateral toral nucleus (see R1 in **Figure 2**). In contrast, caudal tectal injections did not label this area, but label is rather seen in intermediate and caudolateral parts of NL (C2 and C3 in **Figure 2**). Further, dorsal DiI injections into the optic tectum (TeO) resulted in ventrally located label in the intermediate and caudal NL (see D2 and D3 in **Figure 2**), but not at the most anterior NL levels (see D1 in **Figure 2**). Finally, ventral tectal DiI injections labeled the most anterolateral NL (V1 in **Figure 2**) slightly more dorsally than rostral tectal injections, with very little, if any, lateral label at intermediate NL levels (V2 in **Figure 2**) and none at caudal NL levels (V3 in **Figure 2**). A charting of these largely non-overlapping labelings in the NL after tectal quadrant injections is shown in the uppermost row of **Figure 2** in corresponding coloration (rostral tectal injection: red, caudal one: light blue, dorsal one: yellow, ventral one: green).

A second series of quadrant injections with a 45° shift relative to those along the main axes just described (see **Figure 1D** for injection sites) largely confirmed the results of the first one. After a rostroventral tectal injection label was seen dorsally at rostral and intermediate levels in the NL (RV1 and RV2 in **Figure 3**), but not at caudal levels (RV3 in **Figure 3**). A caudoventral tectal DiI injection yielded no label in the anterior NL (CV1 in **Figure 3**), but did so strongly at intermediate and caudal levels in the dorsolateral NL (CV2 and CV3 in **Figure 3**). A rostrodorsal tectal injection resulted in ventral label at anterior and intermediate NL levels (RD1 and RD2 in **Figure 3**), but none was seen at the caudal level (RD3 in **Figure 3**). Finally, a caudodorsal tectal injection lead to no label at anterior and intermediate NL levels (CD1 and CD2 in **Figure 3**), but labeled strongly the ventral NL at caudal levels (CD3 in **Figure 3**). Again, these labelings in the NL are charted with different colors in the uppermost row of **Figure 3** (rostroventral injection: red, caudoventral one: light blue, rostrodorsal one: yellow, caudodorsal one: green).

**FIGURE 4:**
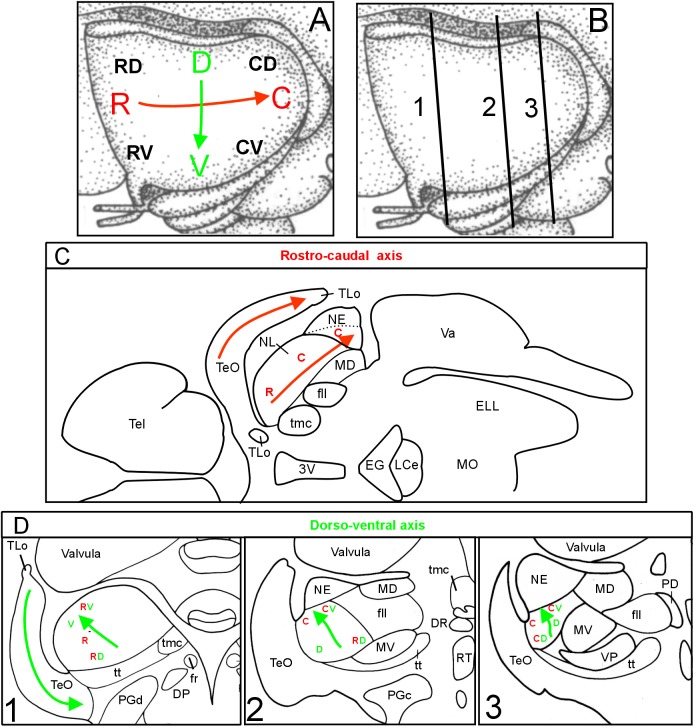
Topographical axes in optic tectum and torus semicircularis. **(A)** Schematic depiction of tectal rostrocaudal (R-C red) and dorsoventral (D-V green) axes and their corresponding topographic representations in the lateral toral nucleus in the brain of *Gnathonemus petersii* as visualized after two series of four DiI tectal quadrant injection sites (see **Figure 1B**), the first series corresponding to the main axes shown in **(A)**, the second series are injections shifted by 45° (also shown in **A** with smaller black letters). **(B)** Three transverse midbrain levels documented in **Figures 1** through 3 which were studied for tecto-toral interconnections. **(C)**: Horizontal section through elephant-nose fish brain at the level of NL shows that the main tectal rostrocaudal axis also runs rostrocaudally within the NL, i.e., is not inverted (brain outline redrawn after Bell and Szabo, 1986). **(D)**: Investigated transverse section levels of the elephant-nose fish brain show that the main dorsoventral tectal axis (indicated by V and D) runs from caudoventral to anterodorsal within the NL, i.e., it is inverted (see green arrows in **D**). Similarly, also the shifted injection sites (indicated by RV, RD, CV, CD) show that the ventral tectum is represented dorsally in the NL and vice versa. In addition, these shifted tectal injections also show that the rostral tectum is rather rostrally and the caudal tectum rather caudally represented in the NL confirming that the rostrocaudal axis is not inverted. See text for details. C, caudal; CD, caudodorsal; CV, caudoventral; D, dorsal; DP, dorsal posterior thalamic nucleus; DR, rostrodorsal tegmental nucleus; EG, eminentia granularis; ELL, electrosensory lateral line lobe; fr, fasciculus retroflexus; fll, lateral longitudinal fascicle; LCe, lobus caudalis cerebelli; MD, mediodorsal nucleus of torus semicircularis; MO, medulla oblongata; MV, medioventral nucleus of torus semicircularis; NE, exterolateral nucleus of torus semicircularis; NL, lateral nucleus of torus semicircularis; PD, dorsal perilemniscal part of nucleus lateralis valvulae; PGd, dorsal preglomerular nucleus; PGc, caudal preglomerular nucleus; R, rostral; RD, rostrodorsal; RV, rostroventral; RT, rostral tegmental nucleus (of Grover and Sharma, 1981); Tel, telencephalon; TeO, tectum opticum; TLo, torus longitudinalis; tmc, mesencephalo-cerebellar tract; tt, toro-pre-eminential tract; V, ventral; Va, Valvula cerebelli; VP, ventroposterior nucleus of torus semicircularis; 3V, third ventricle.

We conclude from these tracing experiments that there is a clear reciprocal topographical relationship between the TeO and the NL. The rostrocaudal tectal axis (red arrow in **Figure 4A**) is paralleled by a rostrocaudal representation in the lateral toral nucleus (red arrows in TeO and NL in **Figure 4C**). This conclusion follows clearly from the positions of the red R and C letters at three investigated NL levels, which distribute in a rostrocaudal pattern in both series of experiments (**Figure 4D**). Furthermore, the tectal dorsoventral axis (green arrow in **Figure 4A**) runs orthogonally to the rostrocaudal one, that is, from caudoventral levels toward anterodorsal levels within the NL (green arrows between V and D in **Figure 4D**) and is thus inverted with regard to the tectal dorsoventral axis. For the sake of simplicity, we use ventral and dorsal in the brain identical as for the general body axis (see discussion in Herget et al., 2014). This inversion is more difficult to grasp because it also involves a rostrocaudal gradient, but is nevertheless clearly seen by looking at the location within the NL of green V’s (indicating a ventral tectal representation) and D’s (indicating a dorsal tectal representation) after the first series of injections along the main tectal axes (**Figure 4D**). Plotting the locations of tectal label within the NL after rostroventral (RV), caudoventral (CV), rostrodorsal (RD) and caudodorsal (CD) injections the picture is similar: also after these injections V’s lie always dorsal, D’s ventral in the NL at all three toral levels.

#### Connections With Remaining Toral Nuclei

**FIGURE 5:**
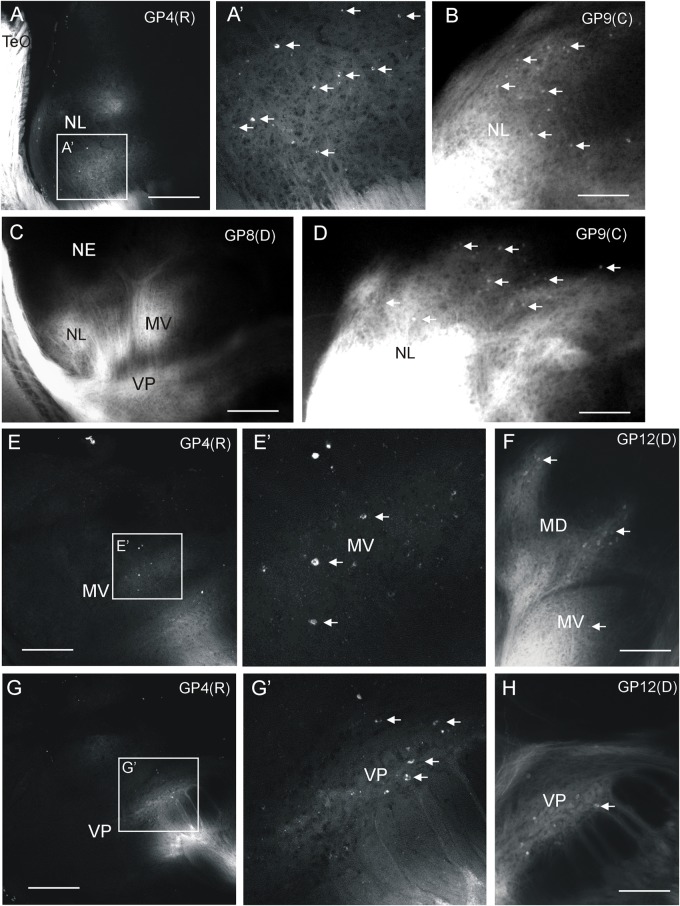
Demonstration of ipsilateral retrograde and anterograde label in torus semicircularis after DiI injections into optic tectum of *Gnathonemus petersii* shown in confocal optical sections **(A,E,G)** and epifluorescent microscopical microphotographs **(B,C,D,F,H)**. Lateral is to the left. White **arrows** indicate retrogradely labeled cells (note contrast between black cell nucleus and white cytoplasm in confocal pictures). **(A)** Confocal overview of the lateral toral nucleus after rostral tectal injection and **(A’)** detailed enlargement showing labeled terminals and cell bodies. **(B,D)** Two examples of epifluorescent microphotographs showing retrogradely labeled cells at the edge of the labeled field in the lateral toral nucleus after a caudal tectal injection. **(C)** Overview shown in an epifluorescent microphotograph with labeled fields in the lateral, medioventral and ventroposterior toral nuclei after a dorsal tectal injection. **(E)** Confocal overview of the medioventral toral nucleus after a rostral tectal injection and **(E’)** detailed enlargement showing labeled terminals and cell bodies. **(F)** Epifluorescent microphotograph shows anterograde and retrograde label in mediodorsal and medioventral toral nuclei after a dorsal tectal injection. **(G)** Confocal overview of the ventroposterior toral nucleus after a rostral tectal injection and **(G’)** detailed enlargement showing labeled terminals and cell bodies. **(H)** Epifluorescent microphotograph shows antero- and retrograde label in the ventroposterior toral nucleus after a dorsal tectal injection. Size bar in **(A,C,E,G)**: 0,5 mm. Size bar in **(B,D,F,H)**: 0.25 mm. See text for details. GP4(R), *G. petersii* 4 with rostral tectal injection; GP8(D), *G. petersii* 8 with dorsal tectal injection; GP9(C), *G. petersii* 9 with caudal tectal injection; GP12(D), *G. petersii* 12 with dorsal tectal injection; MD, mediodorsal nucleus of torus semicircularis; MV, medioventral nucleus of torus semicircularis; NE, nucleus exterolateralis of torus semicircularis; NL, nucleus lateralis of torus semicircularis; TeO, tectum opticum; VP, ventroposterior nucleus of torus semicircularis.

Additional label after all tectal DiI injections was seen in the medioventral (MV) and ventroposterior toral (VP) nuclei, but never in the exterolateral nucleus (NE) (**Figures 2**, **3**). Unequivocal label in the mediodorsal toral (MD) nucleus was only seen after ventral tectal injections. In all four labeled toral nuclei (NL, MD, MV, VP) we saw evidence for anterograde and retrograde labeling. Despite the heavy DiI labeling in toral nuclei evident in epifluorescent microscopical sections after tectal tracer injections, it was still possible to discern retrogradely labeled cell bodies at the border of labeled DiI fields (arrows in **Figures 5B,D,F**). However, in order to ensure that we see retrogradely labeled cell bodies as well as terminal fields throughout the labeled fields in the torus semicircularis after tectal tracer injections, we investigated some experimental cases with confocal microscopy. This confocal analysis clearly revealed more extensively retrogradely labeled cell bodies (see arrows in **Figure 5**) in the lateral (**Figure 5A,A**’), medioventral (**Figure 5E,E**’) and ventroposterior nuclei (**Figure 5G,G**’).

#### Additional Tectal Connections

**FIGURE 6:**
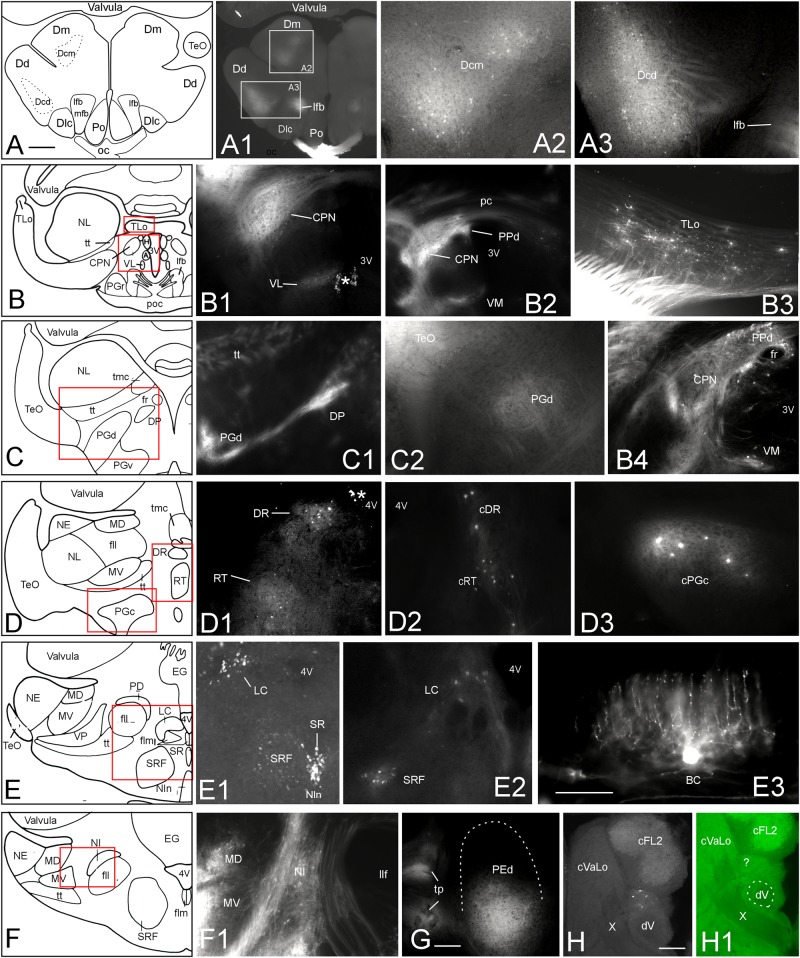
Additional tectal connections after DiI injections into the optic tectum of *Gnathonemus petersii*. Generally, connections are ipsilateral and lateral is to the left. Contralaterally labeled structures are indicated with prefix “c”. **(A–F)** Drawings of brain cross sections with areas shown in microphotographs **(A1)** through **(F1)** highlighted by red or white rectangles. **(A1,A2)** Telencephalic level caudal to anterior commissure. **(A2)** Medial part of central zone of dorsal telencephalon. **(A3)** Dorsal part of central zone of dorsal telencaphalon. **(B1)** Central pretectal nucleus (CPN) and ventrolateral thalamic nucleus (VL). **(B2)** Central and dorsal periventricular pretectal and ventromedial thalamic nuclei. **(B3)** Torus longitudinalis. **(B4)** Central and dorsal periventricular pretectal and ventromedial thalamic nuclei with emphasis on retrogradely labeled cells. **(C1)** Dorsal preglomerular and dorsal posterior thalamic nuclei. **(C2)** Dorsal preglomerular nucleus. **(D1)** Dorsal reticular and rostral tegmental nuclei. **(D2)** Same nuclei contralaterally. **(D3)** contralateral caudal preglomerular nucleus. **(E1)** Superior raphe, superior reticular formation and Locus coeruleus. **(E2)** Locus coeruleus and superior reticular formation. **(E3)** Valvular basal efferent cell. Note extensive dendritic tree. **(F1)** Nucleus isthmi and mediodorsal and medioventral toral nuclei. **(G)** Dorsal pre-eminential nucleus. **(H)** Labeled neurons ventral to cFL2 and lateral to vagal lobe. Note that this section level is indicated in **Figure 8Q**. **(H1)** Microphotograph as **(H)** in green epifluorescence to highlight entrance of vagal nerve. Asterisk: artifact. Size bar in **(A)**: 0.5 mm, applies to **(A1)** and **(B)** through **(F)**. Size bars in **(E3,G,H)**: 0.25 mm. See text for details. A, anterior thalamic nucleus; BC, basal (efferent cerebellar) cell; cDR, contralateral DR; cFL2, contralateral lateral funicular nucleus 2; cPGc, contralateral PGc; CPN, central pretectal nucleus; cRT, contralateral RT; cVaLo, contralateral vagal lobe; dV, descending trigeminal root; Dcd, Dcm, dorsal and medial parts of central zone of dorsal telencephalon; Dd, dorsal zone of dorsal telencephalon; Dlc, part c of lateral zone of dorsal telencephalon; Dm, medial zone of dorsal telencephalon; DP, dorsoposterior thalamic nucleus; DR, rostrodorsal tegmental nucleus; EG, eminentia granularis; fll, lateral longitudinal fascicle; flm, medial longitudinal fascicle; fr, fasciculus retroflexus; H, habenula; LC, locus coeruleus; lfb, lateral forebrain bundle; MD, mediodorsal nucleus of torus semicircularis; mfb, medial forebrain bundle; MV, medioventral nucleus of torus semicircularis; NE, exterolateral nucleus of torus semicircularis; NI, nucleus isthmi; Nln, nucleus interpeduncularis; NL, lateral nucleus of torus semicircularis; oc, optic chiasma; pc, posterior commissure; PD, dorsal perilemniscal part of nucleus lateralis valvulae; PEd, dorsal pre-eminential nucleus; PGc, PGd, PGr, PGv caudal, dorsal, rostral, ventral preglomerular nuclei; Po, preoptic region; poc, postoptic commissure; PPd, dorsal periventricular pretectal nucleus; RT, rostral tegmental nucleus (of Grover and Sharma, 1981); SR, superior raphe; SRF, superior reticular formation; TeO, tectum opticum; TLo, torus longitudinalis; tmc, mesencephalo-cerebellar tract; tp, tecto-pre-eminential tract; tt, toro-pre-eminential tract; VL, VM ventrolateral, ventromedial thalamic nucleus; VP, ventroposterior nucleus of torus semicircularis; X, vagal nerve; 3V, third ventricle; 4V, fourth ventricle.

To further corroborate tectal connectivity seen in our DiI tracings with previous results (Wullimann and Northcutt, 1990), we document here additional labeled structures outside of the torus semicircularis after tectal DiI injections. In general, label was stronger ipsilateral than contralateral. After 10 weeks of incubation we saw retrogradely labeled neurons in the central division of the dorsal telencephalic area (Dcm, Dcd) posterior to the anterior commissure (**Figures 6A–A3**). In the diencephalon, we noted retrograde (and likely anterograde) label in the dorsal periventricular pretectal (PPd) and central pretectal nuclei (CPN), as well as in the ventromedial and ventrolateral thalamic nuclei (VM, VL) (**Figures 6B–B2,B4**), and furthermore in the ipsilateral torus longitudinalis (TLo) (**Figure 6B3**). Also, retrograde and anterograde label was seen in the ipsilateral dorsal posterior thalamic nucleus (DP) (**Figures 6C–C1**) and only anterograde label in the dorsal preglomerular nucleus (PGd) (**Figures 6C–C2**). More caudally, we saw bilateral retrograde label in the dorsorostral tegmental nucleus (DR) and the rostral tegmental nucleus of Grover and Sharma (RT) (1981) (**Figures 6D–D2**) and only contralaterally in the caudal preglomerular nucleus (PGc) (**Figure 6D3**). Furthermore, cell bodies were labeled in the locus coeruleus (LC), superior raphe (SR) and superior reticular formation (SRF) (**Figures 6E–E2**). Finally, labeled cell bodies and fibers were present in the ipsilateral nucleus isthmi (NI) (**Figures 6F,F1**). More caudally, we also always saw antero- and retrograde label in a part of the ipsilateral dorsal pre-eminential nucleus (PEd) after tectal injections (**Figure 6G**). Moreover, a few retrogradely labeled cells were seen in the lateral part of the primary mechanosensory lateral line medial octavolateralis nucleus (MON, not shown). A few scattered retrogradely labeled cells were seen contralaterally in the anterior rhombencephalon possibly in a part of the descending trigeminal nuclear complex (**Figures 6H,H1**). These cells were located between the lateral funicular nucleus 2 dorsally and the descending trigeminal root ventrally (following Amey-Özel et al., 2015). The labeled cells layed at the level of the entrance of the vagal nerve (level is indicated in **Figure 8Q**; after Lázár et al., 1992). Also, descending tecto-bulbar fibers – which partially crossed in the ansulate commissure – were observed (not shown). These data, as well as those on toral connections with the optic tectum, are entirely confirmatory to an earlier study using horseradish peroxidase tracing (Wullimann and Northcutt, 1990) except for the label in the caudal preglomerular, the pre-eminential and the medial octavolateralis nuclei as well as in the descending trigeminal nuclear column (see Discussion).

#### Tectal Connections With the Valvula Cerebelli

**FIGURE 7:**
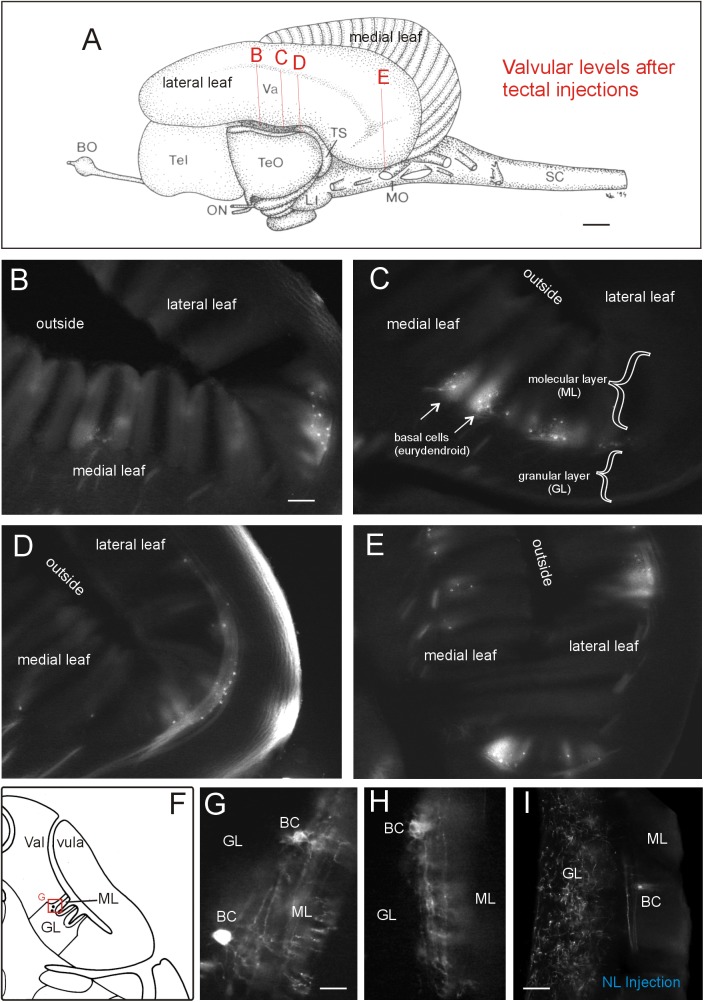
Valvular projections to optic tectum in *Gnathonemus petersii*. **(A)** Lateral view of the brain of the elephant-nose fish *G. petersii* highlighting extent of retrograde label in valvula after a tectal DiI injection by indicating levels of sections shown below. **(B–E)** Levels from rostral to caudal with retrograde label of basal efferent (eurydendroid) cells (**arrows**). Note that there are no terminals in the granular cell layer, i.e., there is no anterograde (mossy fiber type) label. **(F)** Drawing of a cross-sectioned cerebellar valvula of the elephant-nose fish explaining position of retrogradely labeled basal efferent (eurydendroid) cells. **(G,H)** two higher-power examples of retrogradely labeled basal (eurydendroid) cells after tectal DiI injections. Note that an extensive dendritic tree is labeled extending into the molecular layer. **(I)** Example of valvular retrograde and anterograde label after a DiI injection into the lateral toral nucleus. Note that in this case also mossy fibers are labeled in the granular layer in addition to basal (eurydendroid) cells. Size bar in **(A)**: 1 mm. Size bar in **(B)**: 0.2 mm, also applies to **(C)** through **(E)**. Size bar in **(G,H)**: 0.05 mm. Size bar in **(I)**: 0,1 mm. See text for details. BC, basal (efferent cerebellar) cells; BO, bulbus olfactorius; GL, cerebellar granular layer; LI, lobus inferior; ML, cerebellar molecular layer; MO, medulla oblongata; NL, lateral nucleus of torus semicircularis; ON, optic nerve; SC, spinal cord; Tel, telencephalon; TeO, tectum opticum; TS, torus semicircularis; Va, valvula cerebelli.

A rather large extent of the cerebellar valvula (see indicated levels in **Figure 7A** and corresponding microphotographs) exhibited retrogradely labeled efferent basal cells after tectal DiI injections. An exemplary retrogradely labeled tectopetal valvular basal efferent cell body including its extensive dendritic tree is shown in **Figure 6E3**. However, in contrast to lateral toral nucleus DiI injections (see below), label after tectal injections was strictly ipsilateral and furthermore did not extend into the most rostral valvula covering the telencephalon. The labeled cells are called basal cells in the valvula (Nieuwenhuys and Nicholson, 1969b) and correspond functionally to eurydendroid cells in the teleostean corpus cerebelli. Labeled basal cells were seen in medial as well as lateral valvular leafs (**Figures 7B–E**). Basal cells were easily identified because they layed typically at the base of a leaflet of molecular layer in a position intermediate to molecular and granular layers (see explanatory drawing in **Figure 7F**). High power microphotographs revealed details of basal cell labeling and show in addition to the cell soma an extended dendritic tree in the molecular layer (**Figures 7G,H**). As there was never anterograde label in the granule cell layer, the optic tectum evidently received only afferent input from the valvula and did not project to it (as opposed to the lateral toral nucleus, see next paragraph).

#### Injections Into the Lateral Toral Nucleus

**FIGURE 8:**
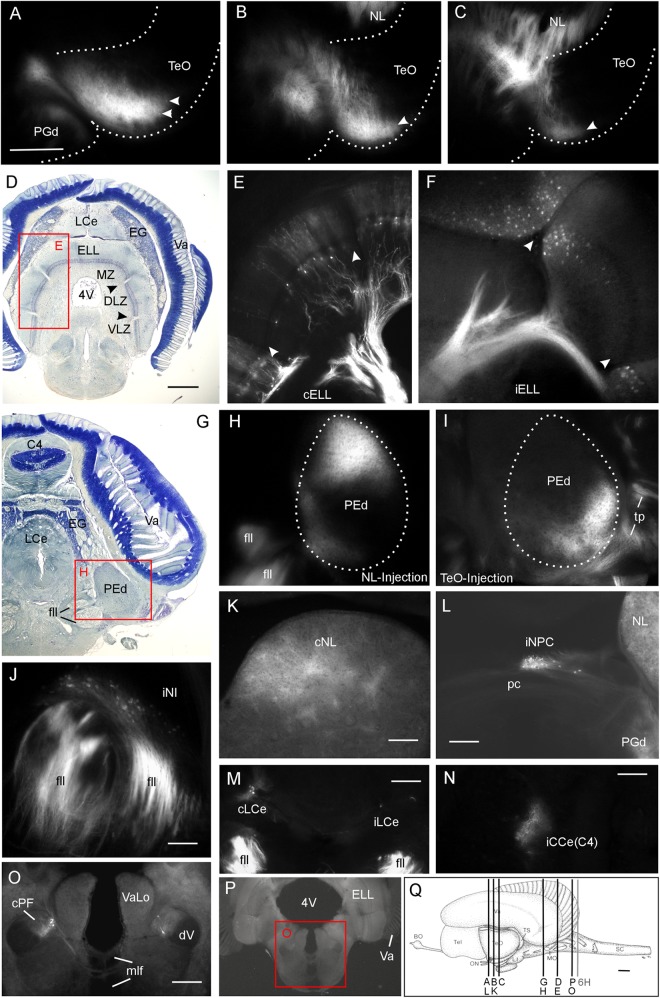
Lateral toral nucleus (NL) connections after a dorsally positioned DiI injection in *Gnathonemus petersii* (see **Figure 1C**). **(A–C)** Retro- and anterograde label in the ipsilateral most rostroventral optic tectum. **Arrowheads** in **(A–C)** point to retrogradly labeled cells. **(D)** Bodian-Cresyl stained section at the level of the electrosensory lateral line lobe (ELL). **(E,F)** Retrogradely labeled cells in ventral aspect of the medial zone and dorsal label in dorsolateral and ventrolateral zones (see text for more). Note stronger contralateral **(E)** and weaker ipsilateral label **(F)** in the ELL. **Arrowheads** in **(D–F)** point to glial boundaries between three zones. **(G)** Bodian-Cresyl stained section at the level of the dorsal pre-eminential nucleus. **(H)** Note that only the dorsal part of PEd is labeled. **(I)** In comparison, the ventral part of the PEd is labeled after a caudal tectal injection. This shows that both the NL and the optic tectum are reciprocally interconnected with the dorsal pre-eminential nucleus. **(J)** Retrograde label in nucleus isthmi. **(K)** The most dorsal part of NL is labeled contralaterally representing an inverted image of the ipsilateral injection site. **(L)** Retrograde label in the ipsilateral nucleus paracommissuralis. **(M,N)** Retrograde label in the caudal cerebellar lobe and the cerebellar corpus, respectively. **(O)** Retrograde label in a hindbrain nucleus belonging to the trigeminal sensory column, i.e., the funicular part of the descending trigeminal nucleus (after Amey-Özel et al., 2015). **(P)** Overview shows location of **(O)**. **(Q)** Schema shows section levels of label shown in this figure and of **Figure 6H**. Size bar in **(A)**: 0.5 mm, also applies to **(B,C)**. Size bar in **(D)**: 0.5 mm, also applies to **(G)**. Size bars in **(K)** through **(N)** and **(P)**: 0.25 mm. Size bar in **(Q)**: 1 mm. See text for details. BO, bulbus olfactorius; C4, C4 lobe of corpus cerebelli; CCe, corpus cerebelli; cELL, contralateral ELL; cNL, contralateral NL; cPF, contralateral funicular part of descending trigeminal nucleus; dV, descending trigeminal root; DLZ, dorsolateral zone of ELL; EG, eminentia granularis; ELL, electrosensory lateral line lobe; fll, lateral longitudinal fascicle; iCCe, ipsilateral CCe; iELL, ipsilateral ELL; iLCe, ipsilateral LCe; iNI, ipsilateral nucleus isthmi; iNPC, ipsilateral NPC; LCe, lobus caudalis cerebelli; LI, lobus inferior; mlf, medial longitudinal fascicle; MO, medulla oblongata; MZ, medial zone of ELL; NI, nucleus isthmi; NL, lateral nucleus of torus semicircularis; NPC, nucleus paracommissuralis; ON, optic nerve; pc, posterior commissure; PEd, dorsal pre-eminential nucleus; PGd, dorsal preglomerular nucleus; SC, spinal cord; Tel, telencephalon; TeO, tectum opticum; tp, tecto-pre-eminential tract; TS, torus semicircularis; Va, valvula cerebelli; VaLo, vagal lobe; VLZ, ventrolateral zone of ELL; 4V, fourth ventricle.

We furthermore injected the lateral toral nucleus (NL) using a dorsal approach either removing the valvula dorsal to the left torus semicircularis or, alternatively, most of the left optic tectum (TeO). In both cases, a DiI injection was placed in the center of the dorsal aspect of the lateral toral nucleus (**Figure 1C**). The connections were largely confirmatory to previous studies (summarized in Wullimann and Grothe, 2013). Importantly, since the anterodorsal aspect of the lateral toral nucleus was not labeled after any of the four tectal quadrant injections along the major two tectal axes (**Figure 2**), but was labeled after a rostroventral tectal injection, we were particularly keen on seeing where the label is located in the TeO after anterodorsal NL injections. We found strong ipsilateral (and weak contralateral) antero- and retrograde label exclusively in the most rostroventral division of the TeO (**Figures 8A–C**; arrowheads indicate retrogradely labeled cell bodies). The label was within the superficial fibrous and gray layer below the marginal tectal layer. As can be seen from these section levels (compare **Figures 8A–C** with **Figure 8Q**), the label did not extend into the caudal half of the TeO. Thus, the NL tracer injections confirmed the picture gained by the tectal injections. However, the fact that we did not see contralateral connections after tectal injections remains enigmatic.

**FIGURE 9:**
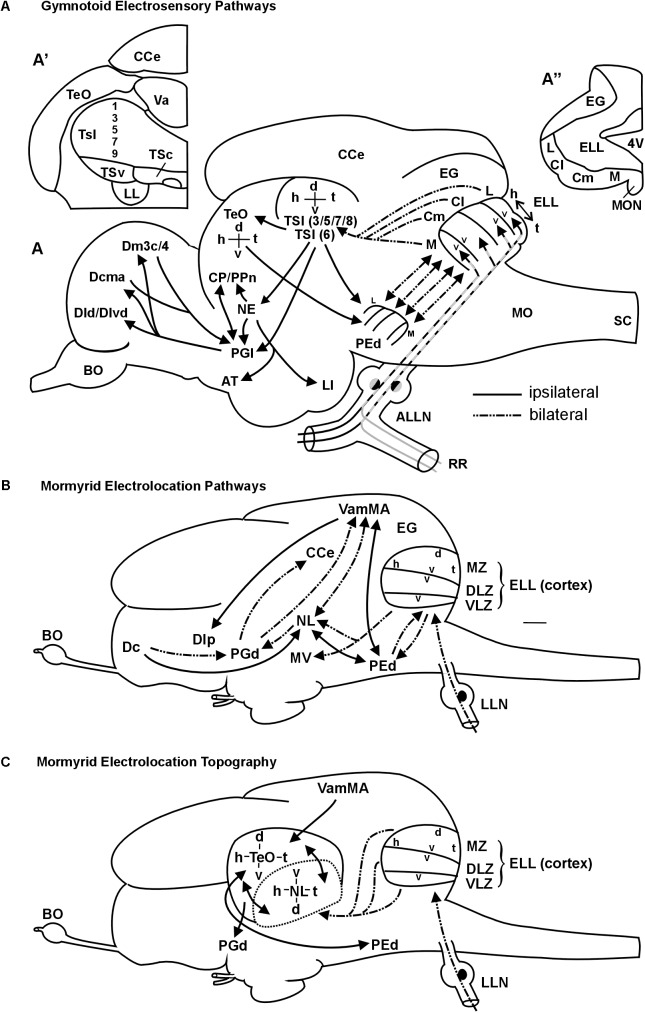
Summary schemes of ascending teleostean electrosensory pathways (modified after Wullimann and Grothe, 2013). **(A)** South American knifefishes or gymnotoids (for citations see text). Note that each of the four segments of electrosensory lateral line lobe and pre-eminential nucleus contains a map of the fish’s entire electrosensory body surface (h, head; t, tail) with alternating dorsoventral orientation (v, ventral; d, dorsal). Tuberous organ input reaches layers 3,5,7,8 (P-units) and layer 6 (T-units) (see text for more). **(A’)** shows one midbrain side with toral divisions in transverse section. For space reasons only the odd-numbered layers are indicated. **(A”)** shows one side of the electrosensory lateral line lobe and eminentia granularis with arrangement of body map segments (ampullary organs: represented in medial segment; tuberous organs in remaining segments). The gymnotoid lateral torus semicircularis (TSl) and optic tectum each display a single, merged electrosensory map. **(B)** Elephant-nose fishes or mormyrids (for citations see text). Mormyromast-ampullary organ electrosensory lateral line pathways. Note that each of the three ELL zones contains a complete body map of the electrosensory skin periphery, with differing dorsoventral axes as indicated in the figure (ampullary organs: represented in ventrolateral zone; mormyromast A-type fibers in medial and B-type fibers in dorsolateral zone). The lateral line nerve (LLN) represents both anterior and posterior nerves. **(C)** Topography between mormyrid ELL and TeO as newly established in this study. In addition, the solely afferent tectal connection with the valvula and solely efferent tectal connection to the dorsal preglomerular nucleus, as well as the reciprocal interconnection with the pre-eminential nucleus are shown. See text for more information. ALLN, anterior lateral line nerve; AT, anterior tuberal nucleus; BO, bulbus olfactorius; Cl/Cm, centrolateral, centromedial segment of ELL (tuberous organs); CCe, corpus cerebelli; CP/PPn, central posterior thalamic/prepacemaker nucleus; d, dorsal; Dcma, anterior part of centromedial zone of pallial area dorsalis telencephali; Dld/Dlvd, laterodorsal/dorsal part of lateroventral zone of pallial area dorsalis telencephali; Dm3c/4, two divisions of medial zone of pallial area dorsalis telencephali; EG, eminentia granularis; ELL, electrosensory lateral line lobe; h, head; L, lateral ELL segment (tuberous organs); LI, lobus inferior; LL, lateral lemniscus (lateral longitudinal fascicle); M, medial ELL segment (ampullary organs); MO, medulla oblongata; MON, medial octavolateralis nucleus; NE, nucleus electrosensorius (pretectum); PEd, dorsal pre-eminential nucleus; PGl, lateral preglomerular nucleus; RR, recurrent ramus of ALLN; SC, spinal cord; t, tail; TeO, tectum opticum; TSc/TSl/TSv, central/lateral/ventral nucleus of torus semicircularis; v, ventral; Va, valvula cerebelli. 4V, fourth ventricle, Additional abbreviations for mormyrid brain (B/C): d, dorsal; Dc/Dlp, central/lateroposterior zone of pallial area dorsalis telencephali; DLZ, dorsolateral zone of ELL; EG, eminentia granularis; ELL, electrosensory lateral line lobe; h, head; LLN, lateral line nerves; MV, medial nucleus of torus semicircularis; MZ, medial zone of ELL; NL, lateral nucleus of torus semicircularis; PEd, dorsal pre-eminential nucleus; PGd, dorsal preglomerular nucleus; t, tail; v, ventral; VamMA, medial leaf of valvula cerebelli (mormyromast/ampullary region); VLZ, ventrolateral zone of ELL.

As expected, the ELL was labeled in each of the three separate ventrolateral (VLZ), dorsolateral (DLZ) and medial (MZ) cortical zones after DiI injections into NL; these zones represent ampullary organ, mormyromast B-cell (capacitive, living objects) and A-cell information (resistive, non-living objects), respectively (**Figures 8D–F**). The labeled cells were in the division of each zone that represents the ventral fish body (note that the dorsoventral body axis is inverted in DLZ and VLZ, but maintained in MZ, see also **Figure 9B**). The contralateral ELL side was labeled more strongly than the ipsilateral one. Slightly more anteriorly, the dorsal pre-eminential nucleus (PEd) was situated and was also labeled ipsilaterally (**Figures 8G,H**). A dorsal injection in the NL apparently resulted in dorsal label in the dorsal pre-eminential nucleus. Similarly, after a caudal tectal quadrant injection, also only a part of the PEd was labeled (**Figure 8I**; note that tecto-pre-eminential fibers entered the nucleus from laterally while toro-pre-eminential fibers seen in **Figure 8H** do so from medially). These patchy labeling patterns indicate that both the lateral toral nucleus and the TeO are topographically interconnected with the dorsal pre-eminential nucleus.

Furthermore, eurydendroid cells in restricted locations in the caudal cerebellar lobe (more strongly contraterally) and in the corpus cerebelli (C4, more strongly ipsilaterally) lighted up as retrogradely labeled (**Figures 8M,N**). We also identified retrogradely labeled cells bilaterally in the funicular part of the descending trigeminal nucleus (**Figures 8O,P**). This cross-section level (see **Figure 8Q**) already contained the vagal lobe but was distinctly anterior to that of the cells labeled after tectal injections in the presumptive descending trigeminal nuclear column (see level 6H in **Figure 8Q**). Also of note, the contralateral lateral toral nucleus was labeled anterodorsally, reflecting the position of the ipsilateral injection site (**Figure 8K**). We also noted retrogradely labeled cells ipsilaterally in the central zone of the dorsal telencephalic area and ipsilateral anterograde label in the dorsal preglomerular nucleus (not shown). Somewhat unexpectedly we saw clear retrograde label in the ipsilateral nucleus isthmi after lateral toral nucleus injections (**Figure 8J**) and in what we identify as the diencephalic paracommissural nucleus (NPC) (**Figure 8L**).

Finally, we observed extensive connections of the lateral toral nucleus with the valvula cerebelli (strongly ipsilaterally and weakly on the contralateral side) extending to levels above the telencephalon, and thus exceeding those of the TeO in rostrocaudal extent. Clearly, valvular basal cells were retrogradely labeled. However, also anterogradely labeled mossy fibers terminating in the granule cell layer were visualized (**Figure 7I**). Thus, in contrast to the purely afferent input to the optic tectum from the valvula, the lateral toral nucleus has reciprocal connections with the valvula cerebelli. We did not further document this valvular label after NL tracer injections as it is well described in the literature.

## Discussion

We will first discuss how our present results on tectal and lateral toral nucleus connections in *Gnathonemus petersii* compare to previous studies, followed by a comparative consideration of the neural electrolocation (navigation) pathways in weakly electric fishes of South-America (gymnotiforms) and Africa (mormyriforms). Finally, we look at how the sensory periphery is represented topographically in the CNS in the visual and electrolocation systems of the mormyrid elephant-nose fish.

### Comparison With Other Studies

**Tectal connections** in *G. petersii* have to our knowledge only been reported in one previous study (Wullimann and Northcutt, 1990). As noted in the result section, the present DiI study closely repeats the pattern of labeling reported in this earlier horseradish-peroxidase (HRP) based tracing study. A study on the mormyrid electromotor system confirmed reciprocal connections of the dorsal posterior thalamic nucleus and the ventroposterior toral nucleus with the optic tectum (TeO) (Carlson, 2002). Another study on the mormyrid auditory system showed a projection of the mediodorsal toral nucleus to the TeO (Kozloski and Crawford, 1998). However, the earlier tectal HRP study (Wullimann and Northcutt, 1990) did not investigate topography between torus semicircularis (in particular the lateral toral nucleus) and the TeO, which is the main focus of the present study.

Interestingly, the retrogradely labeled cells in the mediodorsal toral nucleus documented by Wullimann and Northcutt (1990) after a ventral tectal injection fits with our observation of unequivocal label in this toral nucleus only after ventral DiI injections. Moreover, the label in other toral as well as in the remaining brain nuclei and in the valvula cerebelli reported in the present tectal study was similarly already visualized in the HRP study. Beyond this, we here report that after tectal DiI injections retrograde labeling is found ipsilaterally in the pre-eminential nucleus and in very few cells of the MON and contralaterally in a few anterior rhombencephalic cells related to the trigeminal sensory system and in the caudal preglomerular nucleus. This difference is simply due to the fact that the earlier HRP study did not report label posterior to the level of the torus semicircularis and thus does not reflect real differences.

The pattern of afferent inputs to the TeO in *G. petersii* is generally in good agreement with what has been reported in cyprinid (Grover and Sharma, 1981; Luiten, 1981) and percomorph teleost fishes (Northcutt, 1982; summarized in Wullimann, 1997). In these teleosts, there are afferents from nucleus isthmi, locus coeruleus, raphe, reticular formation, torus semicircularis, torus longitudinalis, rostral tegmental nucleus of Grover and Sharma (1981), and, at forebrain levels, from dorsal posterior and ventral thalamic nuclei, as well as periventricular and central pretectal nuclei plus an input from the central zone of the telencephalic dorsal area. However, special for the mormyrid TeO is on the one hand the strong input from the valvula cerebelli – which has never been noted in other teleost species – and on the other hand the reduction of the mormyrid superficial pretectum and the accessory optic system together with the associated tectal connections (see discussion in Wullimann and Northcutt, 1990). Additionally reported tectal inputs in percomorphs from the somatosensory system, such as the primary sensory main and descending trigeminal nuclei (Northcutt, 1982; Xue et al., 2006) and the lateral funicular nucleus (a likely homolog of dorsal column nuclei; Finger, 2000) were far less extensive in *G. petersii*. Furthermore, tectal inputs from the mechanosensory lateral line system (primary sensory MON) reported in the channel catfish (Finger and Tong, 1984) and in the goldfish (McCormick and Hernandez, 1996) or from the perilemniscal nucleus in the sunfish and carp (Northcutt, 1982; nucleus profundus mesencephali in the carp; Luiten, 1981) were also less conspicuous or absent in *G. petersii*.

More recently, information on teleostean tectal connections in an additional teleost group, i.e., salmonids, has been provided on the rainbow trout (Kinoshita et al., 2006). This paper did not report inputs from the telencephalon and from somatosensory or mechanosensory rhombencephalic nuclei, and also no inputs from raphe and locus coeruleus to the tectum, likely due to methodological reasons as all these structures are most remote from the tracer injection site. However, inputs from most other tectum projecting nuclei just mentioned in various teleost species are evident in their chartings of the rainbow trout. Furthermore, Folgueira et al. (2004) have reported an input to the tectum from the central zone of the dorsal telencephalic area in the rainbow trout. An additional input to the salmonid tectum is reported from the region of the nucleus subglomerulosus (their NRLm). The subglomerular nucleus has later been shown to be a likely chemosensory relay nucleus belonging to the posterior tuberculum (Folgueira et al., 2002, 2005). Similarly, in the percomorph tilapia, cells lying immediately dorsal and lateral to the proper hypothalamic corpus mamillare were reported to project to the optic tectum (Sawai et al., 2000). We think that these tectopetal cells lie clearly outside the corpus mamillare and are more likely identifiable as nucleus subglomerulosus. This subglomerulo-tectal projection is in line with the general functional context of a diencephalic relay of ascending sensory information with which the posterior tuberculum (in particular the preglomerular area) is generally involved (Wullimann and Northcutt, 1990; Wullimann and Mueller, 2004; Northcutt, 2006; Vernier and Wullimann, 2009).

Recently, Heap et al. (2018) saw in a Gal4-enhancer trap zebrafish line very rostrally located tectopetal hypothalamic midline neurons and reported an inhibitory influence in deeper tectal layers after optogenetic stimulation paralleled by calcium imaging responses in the optic tectum. These cells lie in the preoptic-hypothalamic area that was recently characterized in the zebrafish with neuropeptides and transcription factor expression (Herget et al., 2014). Tectopetal preoptic cells have been reported previously in carp (Luiten, 1981), channel catfish (Striedter, 1990) and rainbow trout (Kinoshita et al., 2006), but not in the goldfish (Grover and Sharma, 1981) or in the percomorph sunfish (Northcutt, 1982; summarized in Wullimann, 1997). The posteriorly adjacent tuberal hypothalamus has only been reported to project to the tectum in the channel catfish (Striedter, 1990). However, no studies in teleosts reported tectopetal cells in the even more posterior intermediate and caudal hypothalamus. These data altogether suggest that subglomerular as well as preoptic and tuberal hypothalamic input to the optic tectum are not universal features of teleosts. This is also corroborated by the fact that there are no such tectal inputs in the long-nose gar *Lepisosteus osseus*, an ancestral ray-finned fish (Northcutt, 1982). Be that as it may, the optic tectum of *G. petersii* receives neither projections from the subglomerular nucleus nor from any part of the preoptic region or hypothalamus.

Regarding efferent tectal projections in *G. petersii*, there is also considerable agreement with the situation in other teleosts, such as cyprinids and percomorphs as well as salmonids (Kinoshita et al., 2006), for example regarding tectal efferents to dorsal and ventral thalamus, periventricular and central pretectal nuclei, torus semicircularis, reticular formation and nucleus isthmi. Also, tecto-preglomerular projections have been reported in goldfish (Northcutt, 2006) and trout (Folgueira et al., 2005; Kinoshita et al., 2006) similar to the situation in *G. petersii*.

**Lateral toral nucleus connections** in *G. petersii* were initially studied by Curtis Bell and colleagues (Bell et al., 1981; Finger et al., 1981; Bell and Szabo, 1986). As for the tectal connections, our DiI experiments on lateral toral nucleus (NL) connections replicate much of these earlier HRP results and serve as a control of tracing accuracy. In fact, the summary of NL connections from previous studies (**Figure 9B**) may be used to describe what we found. For example, we saw a main input to the contralateral (more strongly) and ipsilateral (more weakly) lateral toral nucleus from all three cortical (ventrolateral, dorsolateral and medial) zones of the ELL, which represent, respectively, mormyromast A and B cell type input as well as ampullary organ input. The NL in turn projects to the dorsal preglomerular nucleus. Also, reciprocal connections with the pre-eminential nucleus were evident. In addition, we document in some more detail the anterograde projection from the NL into the valvular granular layer and the absence thereof from the optic tectum (TeO). In contrast, both the TeO and the lateral toral nucleus are documented here to receive basal efferent cell input from the valvula. This shows that only the lateral toral nucleus has reciprocal connections with the valvula cerebelli and that the TeO exclusively receives input from the valvula. In addition we saw retrograde label in the corpus cerebelli and the caudal cerebellar lobe as well as in the funicular part of the descending trigeminal nucleus (following Amey-Özel et al., 2015), similar to label identified previously in the trigeminal sensory column (Finger et al., 1981).

Beyond this, we report a strictly ipsilateral and retrograde label of a small nucleus dorsal to the posterior commissure, which we tentatively identify as nucleus paracommissuralis. However, this nucleus has been identified so far only in derived teleost taxa, where it typically has a telencephalic input and an output to the cerebellum (Wullimann and Meyer, 1993). There is no evidence for both connections in *G. petersii* of this pretectal tectopetal population (Meek et al., 1986a,b; Wullimann and Northcutt, 1990) and its identification therefore remains doubtful. Surprisingly, we also labeled retrogradely the tectopetal nucleus isthmi in the experiments involving tracer injections into NL, which was not noted in previous studies. In contrast to the labeling of nucleus isthmi after tectal injections, very many fibers are labeled in the lateral longitudinal fascicle after NL injections, which is in agreement with the fact that fibers from the ELL ascend in it only after NL, but not after tectal, injections. In line with the fact that no contamination of the TeO occurred during tracer application, we believe that the nucleus isthmi truly projects also to the NL.

Our NL injections involved the dorsal aspect of the nucleus and the resulting antero- and retrograde label was restricted to the most rostroventral part of the TeO. This is in line with the results of tectal DiI injections into defined quadrants which document the toro-tectal topographical relationship and which show that after a rostroventral tectal injection we see label in NL most dorsally. In addition, the rostroventral tectal injection documented in the earlier HRP study already reported antero- and retrograde label only in the dorsal aspect of the lateral toral nucleus.

### Comparison of Gymnotiform and Mormyriform Electrolocation Pathways

An extensive recent comparative review on the lateral line (including mechanosensory and electrosensory) system in vertebrates is available for detailed information (Wullimann and Grothe, 2013). Here, we focus on gymnotiforms and mormyriforms, the only two teleost groups that emit an EOD and perceive these weak electric signals via their electroreceptors. We will specifically focus on the ascending electrolocation pathways and the maintenance of topography therein. In both gymnotiforms (Maler et al., 1974; Vischer et al., 1989; Lannoo et al., 1989) and mormyrids (Bell and Russell, 1978; Bell, 1981a,b), large hindbrain ELLs are the recipients of primary electrosensory lateral line nerve input (**Figure 9**). In the body periphery, fish of both groups possess ampullary organs and two different types of tuberous receptor organs. Gymnotiforms have P-unit receptors (reflect EOD amplitude) and T-unit receptors (give temporal information; Heiligenberg and Bastian, 1984; Carr and Maler, 1986; Bell and Maler, 2005), whereas mormyrids exhibit knollenorgans and mormyromasts (Bell and Russell, 1978; Bell and Szabo, 1986).

The ELL in both taxa contains topographically organized maps of the sensory periphery within which the head-to-tail representation is always oriented in the same direction, whereas the dorsoventral axis may be inverted (see **Figures 9 A,B**). Ampullary organs are represented in the medial zone in gymnotiforms (Heiligenberg and Dye, 1982; Carr and Maler, 1986; Lannoo et al., 1989) and in the ventrolateral zone in mormyrids (Bell and Russell, 1978; Bell and Szabo, 1986), whereas tuberous organs are represented in the respective remaining maps. Mormyrids have an additional non-topographically organized nucleus of the ELL (not shown), which is devoted to knollenorgan (i.e., electrocommunication) information. In addition, the mormyrid medial and dorsolateral zones process information from A-cells (perception of dead objects) and B-cells (perception of living objects), respectively, both originating in mormyromasts (Bell et al., 1989; von der Emde, 1998), and thus these maps deal with location and identity of objects. In contrast, each receptor cell of a given gymnotiform tuberous organ sends a trifurcated fiber into the lateral, centrolateral and centromedial maps (Carr et al., 1982; Shumway, 1989; Metzner, 1999). From there, P-unit information is relayed to different layers (in particular 3/5/7/8) of the lateral division of the torus semicircularis (TSl) than T-unit information, which reaches deeper layer 6. Gymnotiform ampullary organ fibers reach layers 3 and 7 (**Figure 9A**; Carr et al., 1981; Scheich and Ebbesson, 1981; Maler et al., 1982; Rose and Heiligenberg, 1985; Carr and Maler, 1986; Carr et al., 1986a,b; Rose and Call, 1992). Through radial connections, integration of T-type and P-type information may occur at gymnotiform lateral toral nucleus levels. Importantly, P-type maps converge in the gymnotiform lateral toral nucleus to one single map.

In mormyrids, the three topographical ELL maps converge to one map at midbrain levels (**Figure 9C**). However, their torus semicircularis is not organized into layers, but into distinct nuclei. Thus, electrolocation information (mormyromasts/ampullary organs) reaches the lateral nucleus of the torus semicircularis whereas electrocommunication information (knollenorgans) is processed in a different parallel pathway (Bell et al., 1981; Bell and Szabo, 1986; Grant et al., 1996; Meek et al., 1999; reviewed in Wullimann and Grothe, 2013).

The toral map in gymnotiforms is then projected upon the central layers of the optic tectum (TeO), where it is in register with the visual retino-tectal map localized more superficially (Bastian, 1982; Heiligenberg and Bastian, 1984; Carr and Maler, 1986; Sas and Maler, 1986a,b; Heiligenberg and Rose, 1987). The gymnotiform pre-eminential nucleus is an intermediate link between midbrain (lateral toral nucleus and tectum opticum) and hindbrain, because both midbrain structures project back to the pre-eminential nucleus, which in turn has reciprocal topographical interconnections with the four ELL maps (Carr et al., 1981; Scheich and Ebbesson, 1981; Maler et al., 1982; Sas and Maler, 1983). This descending pathway has been associated with an attentional (searchlight) function (Bastian, 1986a,b; Heiligenberg, 1990; Bell and Maler, 2005).

Similarly in mormyrids, the dorsal pre-eminential nucleus is reciprocally interconnected to both ELL and lateral toral nucleus in topographical fashion (Bell et al., 1981; Finger et al., 1981; Bell and Szabo, 1986; von der Emde and Bell, 1996; Grant et al., 1996; Meek et al., 1999), with a difference to gymnotiforms being that the ELL maps converge already to one map in the pre-eminential nucleus. The neuroanatomical relationship of the mormyrid lateral toral nucleus and optic tectum has not been addressed previously. Our present results suggest that the mormyrid TeO plays an equally elementary role in the processing of electrolocation information as it does in gymnotiforms. Similar to the latter, the mormyrid TeO feeds back to the dorsal pre-eminential nucleus in apparent topographical fashion. More importantly, we here describe a reciprocal topographical interrelationship of the lateral toral nucleus and the optic tectum and therefore a mutual maintenance of toral electrolocation and tectal visual maps in the respective other structure (**Figure 9C**).

Ascending electrosensory circuitry beyond the midbrain is present in both gymnotiforms and mormyrids (**Figures 9A,B**, see Wullimann and Grothe, 2013, for details and citations), but at forebrain levels there is no hard neuroanatomical evidence for topography. Bell and Szabo (1986) documented that small tracer injections into the lateral toral nucleus yielded small terminal fields in the dorsal preglomerular nucleus (their DAP). In the mormyrid pallium, discreet fields for different sensory representations were seen, but no topography was reported (Prechtl et al., 1998; von der Emde and Prechtl, 1999).

The main ascending electrosensory pathway from the midbrain in both fish groups involves the preglomerular complex, through which the telencephalon is reached. Recently, Giassi et al. (2012) reported newly that the gymnotiform lateral and medial preglomerular nuclei are the main relay for electrosensory input from the lateral toral nucleus to various pallial divisions (Dl, Dm), with additional reciprocal connections between the lateral (and medial) preglomerular nucleus and Dc (Corrêa et al., 1998). Wullimann and Grothe (2013) reviewed a more indirect ascending pathway via the nucleus electrosensorius (see **Figure 9A**). Giassi et al. (2012) claim highly hypothetically that the gymnotiform pathway from the lateral toral nucleus to the dorsal preglomerular nucleus carries electrocommunication information from layers 8a/c, because these deeper toral layers are sensitive to EOD frequency differences and modulations. In contrast, the electrolocation pathway, according to their proposal, would run from the lateral toral nucleus via optic tectum to the medial preglomerular nucleus. However, the gymnotiform optic tectum receives input from all lateral toral nucleus layers except layer 1 (Carr and Maler, 1986), thus, including very likely P-type and T-type information. Moreover, although critical for Giassi’s et al. (2012) conclusion, no data are shown for a compartmentalization of inputs from torus and tectum to lateral and medial preglomerular nuclei, respectively. Another problem is that Keller et al. (1990) did not find evidence for tectal input to both preglomerular nuclei after tectal injections in *Eigenmannia virescens*, which is in contradiction to the retrograde label shown by Giassi et al. (2012) after large preglomerular complex injections in *Gymnotus* sp. and may be a species difference or labeling of fibers of passage.

In mormyrids the lateral toral nucleus projects and therefore carries electrolocation information to the dorsal preglomerular nucleus (Finger et al., 1981; Bell and Szabo, 1986) (**Figure 9B**). Peculiar (and different from gymnotiforms) is the heavy involvement of the valvula cerebelli in the mormyrid electrolocation pathway physiologically (Russell and Bell, 1978) and neuroanatomically, in particular regarding a rather indirect ascending pathway from the dorsal preglomerular nucleus via valvula to the pallium (**Figure 9B**; see review Wullimann and Grothe, 2013). In any case, in the mormyrid central, medial and lateral dorsal telencephalic (pallial) zones, largely non-overlapping sensory fields (visual, auditory, mechano- and electrosense) were reported (Prechtl et al., 1998; von der Emde and Prechtl, 1999).

Two important facts relating to the mormyrid dorsal preglomerular nucleus, which receives electrolocation input from the lateral toral nucleus, emerge from the present study: (1) a tectal input is present in addition to the toral input (**Figure 9C**) and (2) both tectal and toral inputs might be topographical as judged from the patchy nature of the inputs. Thus, although this indicates a maintained topography in the dorsal preglomerular area both for tectal and lateral toral nucleus projections, it has not been analyzed in detail. This and a possible topography of the sensory periphery in the pallial telencephalon must be addressed in the future.

### Topography

This section explores how the sensory periphery of the visual and electrolocation (mormyromast) system might converge in the central nervous system of the elephant-nose fish.

The retina of *G. petersii* exhibits throughout its extent grouped modules of more than 300 rods and 25 cones as functional units and it is neither specialized (as a whole or in special retinal parts) for high spatial acuity (cones) nor maximum sensitivity (rods) as it is the case in many other species (Francke et al., 2014). In contrast, retinal receptor cell types in *G. petersii* have discrete intraretinal connections allowing cones (acuity) and rods (sensitivity) and combinations of the two (movement detection) to function complementarily for detecting contrast and moving objects in dimly lit turbid waters (Wagner, 2007; Landsberger et al., 2008; Kreysing et al., 2012).

Lázár et al. (1984) reported that retinal projections reach the entire surface of the contralateral optic tectum (TeO) of *G. petersii* within the stratum fibrosum et griseum superficiale (SFGS), but these projections show a thinner extent caudally than rostrally. Rostrally, there is a patchy termination pattern leaving regularly repeated spots within the SFGS free of retinal input. The topography of retino-tectal projections is regular; that is, no local overrepresentation of any retinal area was seen in an electrophysiological extracellular recording study of response types, shapes and sizes of receptive fields in tectal units (Pusch et al., 2013b).

**FIGURE 10:**
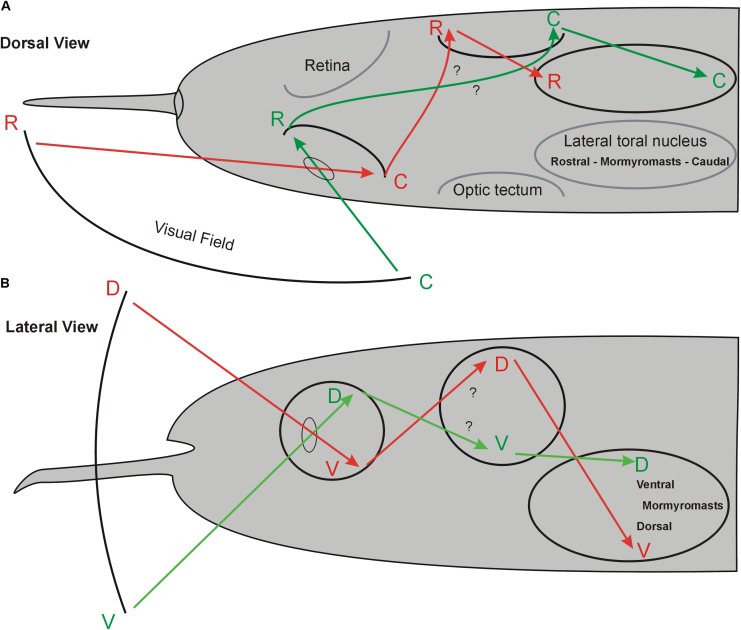
Hypothetical topography of visual field and electroreceptive body surface in the midbrain of *Gnathonemus petersii*. **(A)** Dorsal view. **(B)** Lateral view. The schematics indicate that perception of the visual field of one body side largely overlaps that of the electroreceptive body surface of the same body side in the lateral toral nucleus because both sensory peripheries cross brain sides. Electrosensory topography in the lateral torus nucleus was reported by Hollmann et al. (2016). Note that retino-tectal topography is assumed to be like that shown in many other teleosts (see text) and needs confirmation. See text for more details. C, caudal; D, dorsal; R, rostral; V, ventral.

What about retino-tectal topography? Unfortunately this has not been addressed in mormyrids. However, a comprehensive electrophysiological study on how the visual field is topographically represented in the midbrain TeO was done in various freshwater fishes as diverse as cyprinids (goldfish and carp) and perch-like centrarchids (bluegill sunfish and largemouth black bass) (Schwassman and Kruger, 1965). The authors came to the conclusion that there is a general pattern in all teleosts and stated: “The results show a precisely organized visual projection onto the contralateral tectum in which the anterior visual field lies anteriorly on the tectum, the temporal field posteriorly, the dorsal field medio-dorsally, and the ventral field in the latero-ventral part of the tectum” (cited from Schwassman and Kruger, 1965), thereby corroborating classical neuroanatomical studies on retino-tectal connections in fishes (Lubsen, 1921; Akert, 1949; Leghissa, 1955). Thus, there is good reason to assume that such topographical relationships are also present in *G. petersii* (**Figure 10**). Note that the mormyrid TeO is displaced lateroventrally, so that the mediodorsal tectum becomes the dorsal tectum and the lateroventral tectum the ventral tectum in our **Figure 10**. Also, we call the anterior and temporal visual fields in our figure the rostral and caudal visual fields, respectively.

In contrast to the retinal receptor cells, mormyromasts are distributed unequally on the body surface (Bacelo et al., 2008; Amey-Özel et al., 2015), with the chin appendage, the so-called Schnauzenorgan, and the nasal region particularly densely packed with electroreceptors in comparison to the body trunk surface. Thus, these two regions act as two “foveae” during active electrolocation (von der Emde and Schwarz, 2001; Pusch et al., 2008). Information about the electrically sensed environment enters the (primarily contralateral) lateral toral nucleus (NL) via three separate maps of the primary sensory ELL, which represent mormyromast and ampullary organ body surface information (see above for more details) (Hollmann et al., 2016). These authors furthermore reported that the NL contains a merged map of electroreceptors on the fish body surface that maintains the rostrocaudal body axis but inverts the dorsoventral body axis (see **Figure 10**). Thus, the retinal ganglion cells convey a topographical point-to-point representation of the visual field via the retina to the contralateral TeO. Similarly, the corresponding electrosensory periphery reaches the (primarily contralateral) midbrain lateral toral nucleus through electroreceptors via the ELL.

Our main focus in this contribution is to evaluate whether there is a topographical relationship between the mormyrid optic tectum and lateral toral nucleus and how our present findings on tecto-toral interconnections fit into the picture just described. We found that the tectal dorsoventral axis is - unlike the rostrocaudal axis - inverted anatomically in the lateral toral nucleus (**Figures 4**, **10**). For physical reasons of light passage through the eye, the dorsal visual field is projected upon the ventral retina and the ventral visual field upon the dorsal retina. Furthermore, in agreement with studies discussed above on the general pattern of retino-tectal topography in teleosts, we assume that the mormyrid dorsal and ventral retina project in an inverted fashion to the contralateral ventral and dorsal tectum, respectively. Assuming that the visual fields are largely monocular, it further follows that the rostral visual field is projected upon the caudal (temporal) retina and the caudal visual field is projected upon the rostral (nasal) retina. Again following studies in various other teleosts mentioned above, we assume that the rostral and caudal mormyrid retina then project onto caudal and rostral tectum, respectively (**Figure 10**).

Thus, the ventral tectum “sees” the contralateral ventral visual field and corresponds with its topographical interconnection with the dorsal NL to the latter’s electrosensory (which is predominantly also contralateral) ventral body periphery represented there (Hollmann et al., 2016). In other words, an object in the ventral visual field is “seen” sequentially in the ipsilateral dorsal retina, the ventral contralateral tectum and, finally, in the contralateral dorsal part of the lateral toral nucleus where it converges with the electrolocation information from the contralateral ventral body side sensing the same object as the retina. Likewise, the ventral retina “seeing” an object in the dorsal visual field projects to the contralateral dorsal tectum, the rostral retina (“seeing” a caudal object) projects to the (contralateral) caudal tectum, and the caudal retina (“seeing” a rostral object) projects to the (contralateral) rostral tectum (**Figure 10**). All four tectal quadrants interconnect reciprocally in a topographical fashion with the lateral toral nucleus of the same side (this study) as to overlap there with the contralateral electrosensory body map (Hollmann et al., 2016). In this way, an object close enough to be potentially seen and electrically sensed in any part of the visual field would be represented both visually and electrically (mormyromasts) in the appropriate same location in the lateral toral nucleus (**Figure 10**) and – because of reciprocity of interconnections – also in the appropriate location in the optic tectum.

How could this help in the very specific behavioral assay of cross-modal object recognition as outlined in the Introduction? The fish is confronted to have to recognize a known object with a sensory modality (vision) not used in learning initially the object. Possibly, the fish moves the eyes or body in a way to bring the object onto the NL area where it was initially learned using the electrosense. This might hypothetically be part of the neural process involved in this behavior and is testable by future physiological studies. In this context, the lamprey optic tectum is worth mentioning to highlight an analogous case. As in all vertebrates, the lamprey tectum receives hair cell input from the torus semicircularis (de Arriba Mdel and Pombal, 2007). Moreover, the optic tectum integrates eye and body orientation to allow for locomotion in goal-directed behaviors (Saitoh et al., 2007). Furthermore, the lamprey tectal circuits involved are enhanced by bimodal (visual and electrosensory) activation (Kardamakis et al., 2016).

## Author’s Note

This article is dedicated to Johannes Meek, a pioneer in mormyrid neurobiology.

## Author Contributions

MZ and MW performed the tracing and histology laboratory work. MZ, MW, and GvdE analyzed the data and wrote the manuscript.

## Conflict of Interest Statement

The authors declare that the research was conducted in the absence of any commercial or financial relationships that could be construed as a potential conflict of interest.
